# Social Media Impact on Self-Perceived Oral Health Practices Among Patients Visiting Tertiary Care Hospital in Lucknow: A Cross-Sectional Study

**DOI:** 10.7759/cureus.56206

**Published:** 2024-03-15

**Authors:** Aruna Singh, Gaurav Mishra, Vinay Kumar Gupta, Sumit Kumar, Atrey J Pai Khot

**Affiliations:** 1 Public Health Dentistry, Faculty of Dental Sciences, King George's Medical University, Lucknow, IND; 2 Public Health Dentistry, Goa Dental College and Hospital, Goa, IND

**Keywords:** self-perceived behaviour, social media, oral health care, oral health practices, digital media

## Abstract

Background

Social media is widely used in the medical field, and people often utilize it to learn about their symptoms prior to consulting with a healthcare professional. Hence, the study aims to investigate the influence of social media on self-perceived oral health practices among patients.

Methodology

A cross-sectional study design was adopted, with a questionnaire comprising 15 closed-ended questions. The sample size was estimated to be 451 participants based on the findings from the pilot study. The face validity of the questionnaire was assessed by a subject matter expert (0.83%), and the reliability was measured using Kappa statistics (0.86). The percentile was determined to assess the overall self-perceived oral health practices of the participants. The data was analyzed using descriptive analysis, chi-square test, and multivariate regression analysis at statistical significance (p ≤ 0.05).

Results

The majority of participants belonged to urban areas (66.5%), with an average age of 38.19 ± 6.70. The participants in urban 184 (61.3%) and rural 102 (67.5%) believed that social media provided better knowledge regarding oral health, which was statistically significant (p = 0.046). The majority of the participants, 267 (59.2%), spent more than 30 minutes to three hours per day on social media. It was found that participants who possessed professional occupations had increased odds of having good oral health practices on social media (p = 0.043).

Conclusion

The participants believed that social media provided better knowledge regarding oral health, and self-perceived practices among the participants were found to be poor. Social media platforms provide new educational possibilities in the dentistry sector, but their potential is neglected and unappreciated.

## Introduction

Social media has become an integral component of daily life, frequently outperforming conventional communication channels [1]. Nowadays, social media is seen as a social and cultural change agent that influences how patients and dentists communicate [2]. Although there are several definitions of social media, it is generally agreed upon in the literature that social media facilitates information [3]. The Cambridge Dictionary defines social media as “websites and computer programs that allow people to communicate and share information on the Internet using a computer or mobile phone” [4]. The Internet is home to more than four billion web pages with health-related content [5]. According to research, approximately 75% of individuals globally use the Internet to get health information [6]. Social media use has spread throughout the medical community, and today's patients use it as a source of knowledge to research their symptoms online before consulting with a healthcare professional [7].

The social media platforms let people communicate, express themselves creatively, share their opinions, and learn about health [8]. Users may feel more comfortable expressing their major difficulties since their identities are hidden, their privacy is preserved, and they may engage with material generated by others while looking for health information online [9]. Social media provides oral health care information in different formats for public consumption, including documentaries, blogs, podcasts, tweets, videos, animations, posters, and articles that present oral health care information, often with eye-catching titles [8,9]. These sites may reference reliable material, but they may also concentrate on sensationalized information or utilize information out of context to entice readers [10]. Different social media sites include Facebook (Meta Platforms, Inc., California, USA), YouTube (YouTube, LLC, San Bruno, CA, USA), Twitter (Twitter Inc., San Francisco, CA, USA), Instagram (owned by Meta Platforms, Inc.), and LinkedIn (Microsoft Corporation, Redmond, WA, USA) [11,12]. Furthermore, social media platforms have shown their versatility by providing a diverse range of tools, including interactive blogs and venues for the distribution of audiovisual content that appeals to a large audience that may include future patients [13].

Oral diseases constitute a significant health burden for many countries, impacting over 3.5 billion people globally [14]. A recent study indicated that the general public researches oral health conditions prior to visiting the Oral Health Care Provider (OHCP) [15]. Social media provides people with a simple and effective means to get health information. Social media searches for oral health-related information may help patients feel less anxious, better comprehend existing health issues or treatments, and explore alternatives [12]. However, people may be more inclined to disagree with the diagnosis made by a healthcare provider if they are equipped with false health information from social media. Social media also has a tendency to disseminate false information much more quickly than accurate and verified information, which might lead to cyber chaos or confusion and compromise patient-provider confidentiality, a healthcare practitioner's professional reputation, and licensure difficulties [15,16].

To improve oral health outcomes for patients, OHCPs should educate patients on how to differentiate between evidence-based (EB) health-related information versus non-evidence-based (NEB) health-related information found on social media [17]. Patient decision-making on oral health may be aided by having access to reliable sources of information. It's critical for OHCPs to understand how NEB information may alter patients' opinions about advised care. The concept of dental need is expanded in a significant way by subjective views and perceived needs for dental treatment. The intent of this study is to alert OHCPs to the increasing influence of social media on patients’ perceptions of oral health care. As there is minimal evidence on this topic, the study aims to investigate the influence of social media on self-perceived oral health practices among patients.

## Materials and methods

Study design and study setting

A cross-sectional, questionnaire-based study was conducted among visiting patients in the outpatient department of a tertiary care hospital in Lucknow during July-September 2023. This study followed the Strengthening the Reporting of Observational Studies in Epidemiology (STROBE) guidelines for observational studies.

Ethical consideration, informed consent, and participants recruitment

The study was approved by the Institutional Ethics Committee (Ref. Code: XIX-PGTSC-IIB-IMR-S/P8). The study's objective was conveyed to all participants. The participants had to be active social media users and at least eighteen years old. Study participants voluntarily giving informed consent were recruited in this study. This study complied with the Helsinki Declaration of 1975, revised in 2000, and the ethical guidelines for human experimentation. Those who chose not to provide informed consent and patients with severe disease were excluded from the study.

Sample size and sampling technique

A pilot study was carried out on a sample of 60 patients for pre-testing of the questionnaire to detect any problems with the design, like the ambiguity of words, feasibility, and inability to understand the questions. The questionnaire was further modified based on their feedback. The main study did not incorporate the findings of the pilot trial. The GPower program (G*PowerVersion 3.1.9.4 Statistical Software, Heinrich Heine University Düsseldorf, Düsseldorf) was utilized to compute the sample size for the study at a power of 0.95, an alpha error of 0.05, and data uncured from the pilot study; the sample size was estimated to be 451 [18]. The convenience sampling technique was employed to recruit participants in the study.

Questionnaire design, validation, and data collection

The self-designed questionnaire was developed and comprised of four sections consisting of 15 closed-ended questions. The first section encompassed the collection of demographic information from the participants. The subsequent sections included questions related to behavior, perceptions of patients, and utilization of dental aids by the patients. The questionnaire distributed was in English and the regional language, Hindi. To verify linguistic validity, the English version was back-translated into Hindi and validated by a language expert. An expert committee performed cross-cultural adaptation on the translated version with a view to establishing semantic, idiomatic, experiential, and conceptual similarity between the source and target versions in four domains. Participants with higher education were given the questionnaire in English, while others were given it in Hindi (see Appendix 1, Figure 4). This method guaranteed that the instrument was appropriate for every participant and that the findings were reliable [19]. The face validity of the questionnaire was assessed by a subject matter expert (0.83%). The reliability was measured using Kappa statistics with the resultant Cohen's Kappa coefficient (0.86), indicating a high level of agreement. Participants were instructed to attempt all the questions within 15 minutes. The confidentiality of the information acquired was secured during the study. The criteria for the study were formulated to make it simple and clear. The responses to the questions were summed, and the percentile was determined to assess the overall self-perceived oral health practices of the participants.

Statistical analysis

The recorded data were input into Microsoft Excel (2019; Microsoft Corporation, Redmond, Washington, USA) and analyzed using IBM SPSS® Statistics for Windows, Version 21.0 (IBM Corp., Armonk, NY, USA). Descriptive statistics were presented as frequencies with percentages for categorical variables. The chi-square test was performed for the responses of participants based on self-perceived practices. Multivariate analyses such as multiple linear regression and multivariate logistic regression analysis were performed among the various study variables. The statistical significance was set at P ≤ 0.05.

## Results

Sociodemographic characteristics of participants

The questionnaire was distributed to 460 participants. However, nine of them were incompletely answered and therefore excluded from the study, with a response rate of 98.04%. Most of the participants, 283 (62.7%), were males; more than half of the participants, 276 (61.2%), were unmarried and belonging to urban areas 300 (66.5%). In addition, 437 (96.9%) of the participants were literate with a primary level of education and above. Most of the participants were unemployed, 193 (42.8%) belonging to the lower middle class with an average age of 38.19 ± 6.70. The demographic information of the 451 study participants is shown in Table 1.

**Table 1:** Characteristics of the study participants (N = 451). All values are expressed as the frequency with percentages (in parentheses).

Sociodemographic characteristics		n (%)
Gender	Male	283 (62.7%)
	Female	168 (37.3%)
Marital status	Married	175 (38.8%)
	Unmarried	276 (61.2%)
Area of residence	Urban	300 (66.5%)
	Rural	151 (33.5%)
Education	Primary school	14 (3.1%)
	Middle school	7 (1.6%)
	High school	28 (6.2%)
	Intermediate or post-high school diploma	129 (28.6%)
	Graduate or post-graduate	232 (51.4%)
	Professional degree	41 (9.1%)
Occupation	Unemployed	193 (42.8%)
	Unskilled worker	31 (6.9%)
	Semi-skilled worker	59 (13.1%)
	Skilled worker	74 (16.4%)
	Clerical, shop-owner	43 (9.5%)
	Semi-professional	18 (4.0%)
	Professional	33 (7.3%)
Family income	≤2500	151 (33.5%)
	2501-7439	43 (9.5%)
	7440-12,399	65 (14.4%)
	12,400-18,599	58 (12.9%)
	19,600-24,799	56 (12.4%)
	25,800-49,599	41 (9.1%)
	≥49,600	37 (8.2%)
Total		451

**Figure 1:**
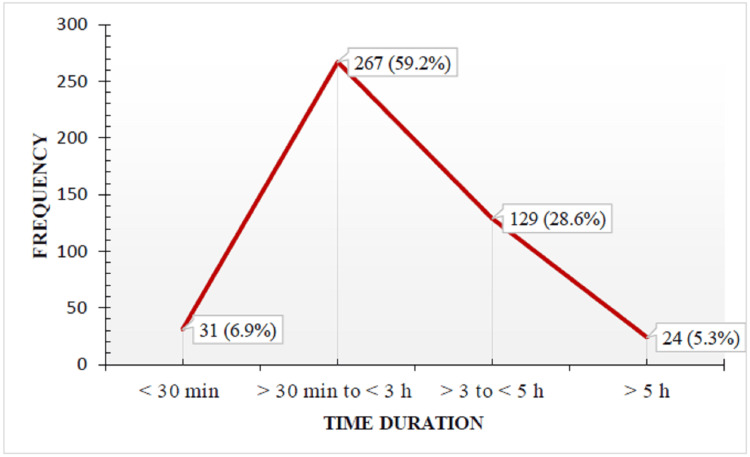
Daily use of social media by participants is shown in a stacked line diagram.

The majority of the participants 267 (59.2%) spent more than 30 minutes to three hours per day on social media, as depicted by the stacked line diagram (Figure 1).

Self-perceived practices of participants towards the use of social media

Table 2 shows the responses of the participants based on their self-perceived practices of using social media. More than half of the participants in urban 184 (61.3%) and rural 102 (67.5%) believed that social media provided better knowledge regarding oral health, which was statistically significant (p = 0.046). The majority of the participants, 225 (75%), followed the correcting brushing technique on social media and admitted that the contents shown on social media promote tobacco consumption in various forms (p≤0.001). Less than half of the participants choose to brush twice daily 180 (39.9%) and use toothbrushes as cleaning aids 201(44.6%) even after the influence of social media. The oral hygiene product advertisements and social media content do not influence the participants to buy the products regardless of belonging to urban or rural (p≤0.001).

**Table 2:** Responses of participants based on self-perceived practices. All values are expressed as the frequency with percentages (in parentheses). Statistical test used: chi-square test. Level of significance: *p ≤ 0.05 is considered statistically significant, **p ≤ 0.001: highly significant association.

Self-perceived practices-based questions	Response	Urban n (%) = 300	Rural n (%) = 151	p-value
Does social media provide you a better knowledge regarding oral health?	Yes	184 (61.3%)	102 (67.5%)	0.046*
	No	116 (38.7%)	49 (32.5%)	
What type of teeth cleaning aids are better for effective cleaning of teeth?	Soft/ultra-soft toothbrush	128 (42.7%)	73 (48.3%)	0.448
	Datun/stick	155 (51.7%)	72 (47.7%)	
	Finger	17 (5.7%)	6 (4.0%)	
Have you ever watched any correct brushing technique content on social media?	Yes	225 (75.0%)	109 (72.2%)	≤0.001
	No	75 (25.0%)	42 (27.8%)	
Who is more reliable regarding oral health concerns like dental aesthetics etc.?	Dentist	211 (70.3%)	101 (66.9%)	0.455
	Information available on social media	89 (29.7%)	50 (33.1%)	
Tooth brushing techniques be learned effectively on social media platforms?	Yes	208 (69.3%)	108 (71.5%)	0.632
	No	92 (30.7%)	43 (28.5%)	
What will you prefer in case of hot/cold sensitivity in tooth?	Visit dentist	108 (36.0%)	49 (32.5%)	0.743
	Take help from social media	169 (56.3%)	89 (58.9%)	
	Ask family/friends	23 (7.7%)	13 (8.6%)	
Do you feel social media content is also one of the reasons for tobacco habit initiation?	Yes	140 (46.7%)	160 (53.3%)	0.025*
	No	59 (39.1%)	92 (60.9%)	
Does social media influence you to brush twice a day?	Yes	117 (39.0%)	63 (41.7%)	0.578
	No	183 (61.0%)	88 (58.3%)	
Does social media content influence you to buy oral hygiene products (toothbrushes/toothpaste/mouthwashes etc.)?	Yes	110 (36.7%)	63 (41.7%)	0.049*
	No	190 (63.3%)	88 (58.3%)	
Does social media influence you for dental flossing?	Yes	197 (65.7%)	92 (60.9%)	0.322
	No	103 (34.3%)	59 (39.1%)	
Does social media influence you for regular use of mouthwashes?	Yes	129 (43.0%)	59 (39.1%)	0.038*
	No	171 (57.0%)	92 (60.9%)	
Does social media content influence you in buying teeth whitening products?	Yes	61 (20.3%)	46 (30.5%)	0.017*
	No	239 (79.7%)	105 (69.5%)	
Do you feel some social media content regarding oral health is unreliable?	Yes	130 (43.3%)	61 (40.4%)	0.552
	No	170 (56.7%)	90 (59.6%)	
Does social media influence you to visit a dentist regularly?	Yes	208 (69.3%)	83 (55.0%)	0.085
	No	92 (30.7%)	68 (45.0%)	
Do you think dentists should respond to the content available on social media platforms?	Yes	163 (54.3%)	78 (51.7%)	0.591
	No	137 (45.7%)	73 (48.3%)	

**Figure 2:**
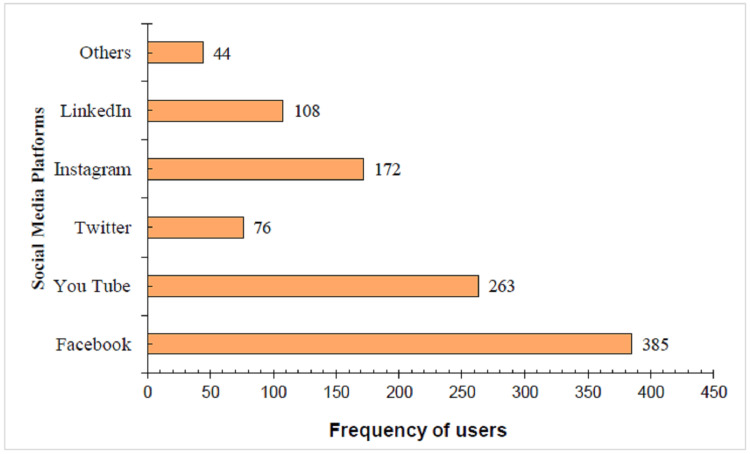
Various social media platforms used by participants.

The most widely preferred platforms by participants are Facebook and YouTube for seeking oral health information (Figure 2).

**Figure 3:**
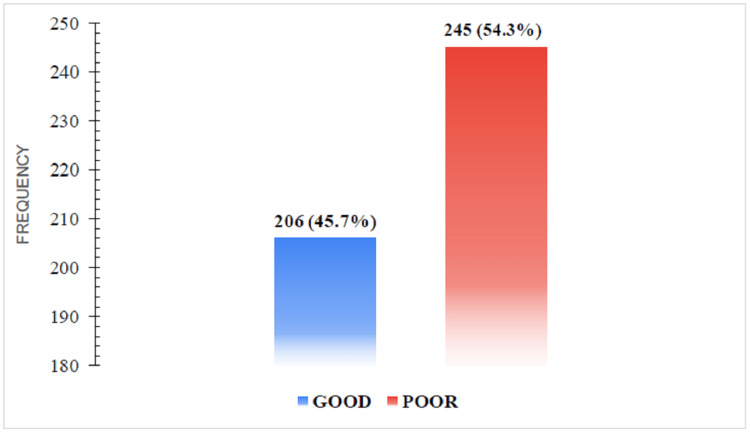
Self-perceived practices among the participants.

**Figure 4:**
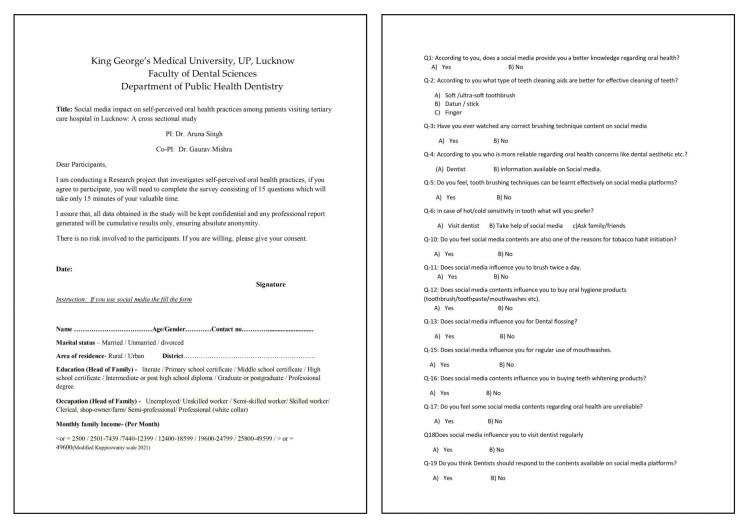
Questionnaire.

Figure 3 illustrates the estimate of the self-perceived practices in percentage among the study participants.

Relationship between self-perceived oral health practices among participants

In the multivariate logistic regression model (category, aOR, 95% CI, P-value), occupation (professional, 3.017 (1.034-0.802), 0.043) showed a statistically significant association with self-perceived practices. It was found that participants who possessed professional occupations had increased odds of having good oral health practices on social media (Table 3).

**Table 3:** Multivariate logistic regression analysis showing predictors of self-perceived practices and independent variables. aOR: adjusted odds ratio; CI: confidence interval. Statistical test used: multivariate logistic regression analysis model; level of significance: *P ≤ 0.05 is considered statistically significant.

Independent variable	Self-perceived practices (excellent/good vs. poor/very poor)			
	B	S.E.	aOR (95% CI)	P–value
Gender				
Male	-	-	1	-
Female	−0.152	0.218	0.859 (0.561–1.316)	0.485
Marital status				
Married	-	-	1	-
Unmarried	−0.493	0.222	0.611 (0.395–0.944)	0.026
Area of residence				
Urban	-	-	1	-
Rural	−0.047	0.216	0.954 (0.624–1.457)	0.827
Education				
Primary school	-	-	1	-
Middle school	1.351	1.238	3.860 (0.341–43.72)	0.275
High school	−0.122	0.702	0.885 (0.224–3.50)	0.862
Intermediate or post-high school diploma	−0.051	0.616	0.950 (0.284–3.176)	0.934
Graduate or postgraduate	−0.681	0.608	0.506 (0.154–1.665)	0.262
Professional degree	−0.194	0.689	0.824(0.213–3.181)	0.779
Occupation				
Unemployed	-	-	1	-
Unskilled worker	−0.022	0.467	0.979 (0.392–2.442)	0.963
Semi-skilled worker	−0.236	0.392	0.790 (0.366–1.702)	0.546
Skilled worker	−0.116	0.391	0.891 (0.414–1.916)	0.767
Clerical, shop-owner	0.534	0.446	1.705 (0.711–4.090)	0.232
Semi-professional	0.085	0.570	1.088 (0.356–3.326)	0.882
Professional	1.104	0.546	3.017 (1.034–8.802)	0.043*
Family income				
≤2500	-	-	1	-
2501-7439	0.138	0.391	1.148 (0.533–2.472)	0.725
7440-12,399	−0.171	0.387	0.842 (0.395–1.798)	0.658
12,400-18,599	0.204	0.411	1.226 (0.548–2.741)	0.620
19,600-24,799	−0.104	0.434	0.902 (0.385–2.109)	0.811
25,800-49,599	−0.616	0.456	0.540 (0.221–1.320)	0.177
>OR = 49,600	−0.624	0.533	0.536 (0.188–1.525)	0.242

Multiple linear regression analysis models showed a statistically significant relationship between practice score with marital status (β = 0.67; 95%CI: 0.24-1.10; P = 0.002), education (β = 0.22; 95%CI: 0.02-0.42; P = 0.034) and occupation (β = −0.20; 95%CI: −0.34 to −0.06); P = 0.005) (Table 4).

**Table 4:** Multiple linear regression on factors associated with self-perceived oral health practices. β: regression coefficient; SE: standard error; CI: confidence interval; the statistical test used: multiple linear regression analysis model; level of significance: *P ≤ 0.05 is considered statistically significant.

Dependent variable	β	SE	t	P-value	95% CI for β	Adjusted R square
Practice score						
Constant	7.153	0.418	17.109	0.000	6.33–7.97	0.047
Gender (ref: male)	0.072	0.218	0.329	0.742	-0.36–0.49	
Marital status (ref: married)	0.671	0.218	3.074	0.002*	0.24–1.10	
Area of residence (ref: urban)	−0.155	0.214	−0.725	0.469	−0.58–0.27	
Education (ref: primary school)	0.220	0.103	2.126	0.034*	0.02–0.42	
Occupation (ref: unemployed)	−0.202	0.071	−2.850	0.005*	−0.34 to −0.06	
Family income	0.130	0.068	1.915	0.056	−0.003–0.263	

## Discussion

The use of social media and Internet access has skyrocketed in recent years. It is still developing and provides valuable avenues for interacting with individuals about issues or events. Social media is utilized in the field of dentistry as well, and dental clinics may benefit from it. Patients' and healthcare professionals' opinions on the advantages of social media use have been divided. Many evaluations of the easily accessible online medical literature have been categorized as lower standards [16,20]. False information spreads via social media far more quickly than accurate information that can be independently verified, which could lead to confusion or chaos online [15]. However, technology might enhance the delivery of healthcare, particularly in the areas of marketing, instruction, dialogue, and patient condition monitoring [21].

Demographic characteristics

Most of the participants, 283 (62.7%), were males, in contrast to studies conducted by Freire et al. [22] and Al-Ansari et al. [23], where most of them were females, 312 (62%) and 712 (75.4%), respectively. More than half of the participants, 276 (61.2%), were unmarried, belonging to urban areas, 600 (66.5%), and unemployed, 193 (42.8%), which could be attributed to the fact that this sector of the population has greater spare time and ease of access to the Internet to be able to surf across social media platforms much more frequently. In addition, 437 (96.9%) of the participants were literate with a primary level of education and above. This was consistent with research by Al Shahrani et al. [24], which showed that 2950 (63.9%) of respondents had earned a graduate degree. Most of the participants belonged to the lower middle class, so it was fitting that they would rely on social media for their dental queries rather than spending time at a dental clinic. It was found that participants who possessed professional occupations had increased odds of having good oral health practices on social media. One of the main factors influencing oral health is educational attainment; those with higher levels of education are more likely to value oral health and practice proper oral hygiene [24].

Use of social media

The majority of the participants, 267 (59.2%), spent more than 30 minutes to three hours per day on social media, with Facebook being the most used social media platform (n = 385) followed by YouTube (n = 263) and the least used being Twitter (n = 76). Freire et al. [22] in his study stated that Instagram was the most followed platform (n = 23), followed by Facebook (n = 15), while the least used were YouTube (n = 2) and Twitter (n = 4). According to reports by Global Stats on social media Stats in India as of December 2023, Facebook has been the most popular platform (78.07%), and its wide audience and reach, high engagement rate, improved analytics, accurate targeting, and affordable advertising rates are considered to be its reasons [25]. Maharani et al. showed that YouTube was the most popular social media for oral health information [26]. The results of the current study were different from those of El Tantawi et al., who found that Saudi teenagers preferred to use Instagram for oral health information (OHI) [27]. This suggests that the way that different social media are used for OHI may vary depending on the nation, culture, or even the stage at which a particular social media becomes popular [28].

Self-perceived practices

More than half of the participants in urban 184 (61.3%) and rural 102 (67.5%) believed that social media provided better knowledge regarding oral health, which was statistically significant (p = 0.046). These results demonstrate the role that social media plays in providing OHI to this group and call attention to the need for dental practitioners to check the accuracy of OHI, which is typically disseminated without proper quality control. The assertion that social media are the most common source of OHI is supported by the data. This is in line with other research that demonstrated how convenient and easily accessible social media and the Internet are on mobile devices, allowing users to access and utilize them whenever they want [29,30].

The majority of the participants, 225 (75%), followed the correct brushing technique on social media. Less than half of the participants choose to brush twice daily 180 (39.9%) and use toothbrushes as cleaning aids 201 (44.6%) even after the influence of social media. According to the study, brushing was positively correlated with the use of social media for OHI. This could imply that people who already exercise self-care, including cleaning their teeth to maintain oral health, are more likely to search for OHI over social media [26].

The study participants acknowledged that social media contents encourage tobacco consumption in different ways (p≤0.001), which is consistent with research from Korea [31] and Taiwan [32] that found higher rates of tobacco users, lifetime smokers, or potential smokers among problematic Internet users. In addition, Al-Ansari et al. [23] reported that in their study of young Saudi adults, participants who rated their dental health as good and who used the Internet sometimes or sporadically scored lower on tobacco use than those who used it problematically. Additionally, it aligns with a study conducted by Iranian university students, which found that smokers had a higher problematic Internet score than non-smokers. However, another study conducted on young Vietnamese participants found no correlation between smoking and the Internet [33].

Regardless of whether they live in an urban or rural area, the participants are not influenced by social media content or advertisements for the purchase of oral hygiene products (p≤0.001). Dental practitioners might need to divert their attention toward such gimmicks for the dissemination of OHI. Providing evidence-based OHI content may be aided by interpreting study findings into plain language and sharing them to grab online users' attention [26]. For this reason, Koumpouros et al. proposed that social media should be useful in marketing because it can help build patients' trust and meet their demands [34]. As Mangold et al. have also shown [35], the relationship between the creator of a healthcare message and the laypeople who read it is dynamic and always evolving. Healthcare practitioners using social media platforms need to exert some degree of control over the material validity and reliability that reaches the general public since misinformation is widespread and has the potential to be fatal. The significance of accurate Internet health information was noted by Bahkali et al. It is in line with published literature and demonstrates comprehension of the state of patients' requirements, making it a powerful tool for enhancing the healthcare system [36].

Limitations and future recommendations

The cross-sectional nature of the current study places limitations on its ability to establish causality; it can only identify relationships that are measurable through longitudinal cohort studies. In addition, a larger proportion of respondents were from urban areas than rural areas, which may lead to bias relating to the use and access to the Internet and social media services among them. Subsequent research endeavors may assess how patients utilize social media as a means of education, to arrange appointments, or to interact with dental practitioners. Clinical evaluation of oral health outcomes could close a knowledge gap on the current population's oral health status as it relates to social media's influence on the marketing of dental products and the distribution of various dental health-related information.

## Conclusions

The participants believed that social media provided better knowledge regarding oral health, and self-perceived practices among the participants were found to be poor. It is advised to use social media effectively to raise awareness of oral health issues across the nation. A greater understanding of self-perceived oral health status could lead to improvements in oral health services and oral health nationally. Social media platforms provide new educational possibilities in the dentistry sector, but their potential is neglected and unappreciated.

## Appendices

Appendix 1 
